# Top down control of spinal sensorimotor circuits essential for survival

**DOI:** 10.1113/JP273360

**Published:** 2017-04-24

**Authors:** Stella Koutsikou, Richard Apps, Bridget M. Lumb

**Affiliations:** ^1^ School of Biological SciencesLife Sciences BuildingUniversity of BristolBristolUK; ^2^ Sensory and Motor Systems Group, School of Physiology, Pharmacology & Neuroscience, Biomedical Sciences BuildingUniversity of BristolBristolUK

**Keywords:** cerebellum, descending control, nociception, freezing behaviour, periaqueductal grey, spinal cord, survival

## Abstract

The ability to interact with challenging environments requires coordination of sensory and motor systems that underpin appropriate survival behaviours. All animals, including humans, use *active* and *passive* coping strategies to react to escapable or inescapable threats, respectively. Across species the neural pathways involved in survival behaviours are highly conserved and there is a consensus that knowledge of such pathways is a fundamental step towards understanding the neural circuits underpinning emotion in humans and treating anxiety or other prevalent emotional disorders. The midbrain periaqueductal grey (PAG) lies at the heart of the defence‐arousal system and its integrity is paramount to the expression of survival behaviours. To date, studies of ‘top down control’ components of defence behaviours have focused largely on the sensory and autonomic consequences of PAG activation. In this context, effects on motor activity have received comparatively little attention, despite overwhelming evidence of a pivotal role for the PAG in coordinating motor responses essential to survival (e.g. such as freezing in response to fear). In this article we provide an overview of top down control of sensory functions from the PAG, including selective control of different modalities of sensory, including proprioceptive, information forwarded to a major supsraspinal motor control centre, the cerebellum. Next, evidence from our own and other laboratories of PAG control of motor outflow is also discussed. Finally, the integration of sensorimotor functions by the PAG is considered, as part of coordinated defence behaviours that prepare an animal to be ready and able to react to danger.

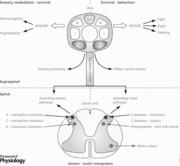

**Abbreviations**
dl: dorsolateral
dl/lPAG: dorsolateral/lateral periaqueductal grey
dm: dorsomedial
l: lateral
PAG: periaqueductal grey
vl: ventrolateral
vlPAG: ventrolateral periaqueductal grey

## Introduction: role of periaqueductal grey in survival

Defence behaviours essential to survival can be innate, but they can also be learnt, and during evolution are conserved across species, including humans (Takahashi, 1992a,b; Blanchard *et al*. 2001a,b; Gross & Canteras, 2012; LeDoux, 2012).

**Figure 1:**
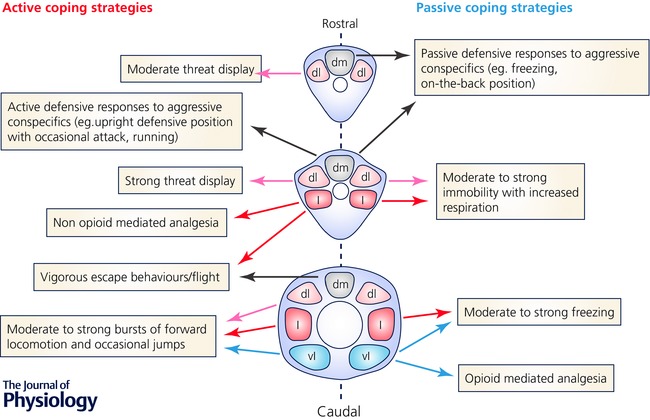
Midbrain periaqueductal grey coordinates emotional defensive behaviours Schematic illustration of the dorsomedial (dm), dorsolateral (dl), lateral (l) and ventrolateral (vl) neuronal columns within the rostral (top of figure) to the caudal (bottom of figure) periaqueductal grey (PAG). Overview of the emotional coping strategies associated with activation of distinct columns of the PAG. The dorsal/lateral columns and the ventrolateral column of the PAG orchestrate active *vs*. passive coping behaviours, respectively. Active coping includes behaviours that are associated with whole body movements, hypertension, tachycardia and a general hyperexcitability. By contrast, passive coping describes a set of behaviours that are generally characterised by lack of movement (but not necessarily flaccid body postures) and decreased responsiveness to the environment (e.g. fear‐evoked freezing mediated by vlPAG activation associated with tense body postures). Information presented in this figure has been collated from experimental work on both cats and rats in the following publications: Bandler & Depaulis (1991), Bandler & Shipley (1994), Bandler *et al*. (2000), Gross & Canteras (2012) and Deng *et al*. (2016).

The midbrain periaqueductal grey (PAG) sits at the heart of the brain circuitry that coordinates survival behaviours and has long been identified as a pivotal component of the so‐called ‘emotional motor system’ (Holstege *et al*. 1996). The PAG surrounds the central aqueduct and, based on patterns of internal and external connectivity and on its cyto‐ and chemo‐architecture, can be divided into four longitudinal columns (dorsomedial (dm), dorsolateral (dl), lateral (l) and ventrolateral (vl); see Fig. 1), each with distinct functions in survival behaviour (Carrive, 1993; Bandler & Shipley, 1994; Behbehani, 1995; Bandler *et al*. 2000; Keay & Bandler, 2002; Tovote *et al*. 2016; Watson *et al*. 2016).

**Figure 2:**
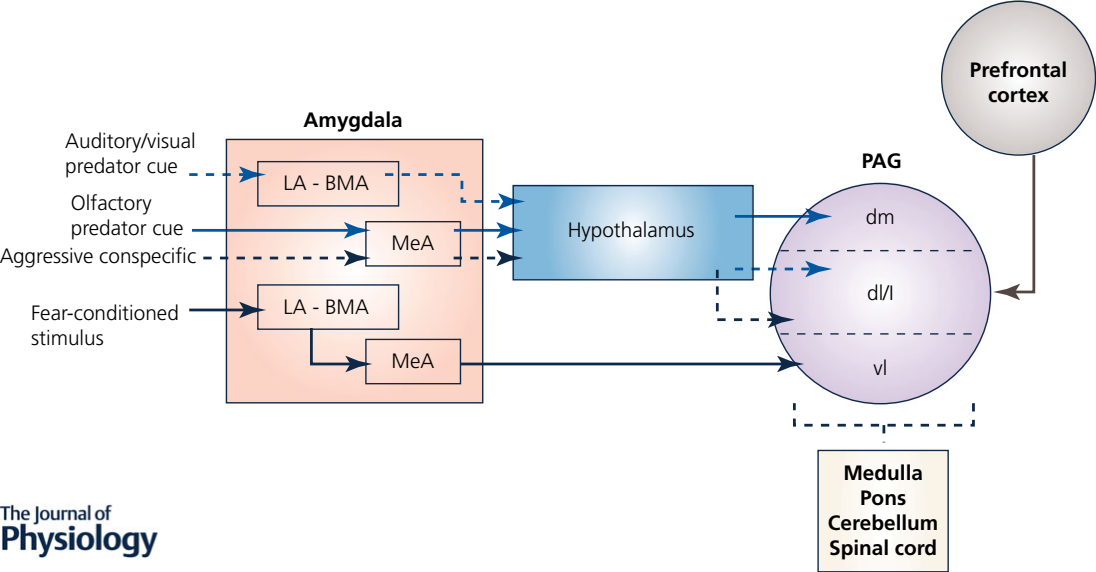
Midbrain periaqueductal grey interactions with the descending limbic system Schematic illustration of the dorsomedial (dm), dorsolateral/lateral (dl/l) and ventrolateral (vl) neuronal columns of the periaqueductal grey (PAG) connections with the limbic system. Efferents from the amydala, hypothalamus and prefrontal cortex target the PAG and, in turn, efferents from the PAG contribute to the descending limbic system that targets medulla, pons, cerebellum and spinal cord. BLA, basolateral amygdala; BMA, basomedial amygdala; CeA, central amygdala; LA, lateral amygdala; MeA, medial amygdala. Information presented in this figure has been collated from the following publications: An *et al*. (1998), Gross & Canteras (2012), Linnman *et al*. (2012), Koutsikou *et al*. (2014) and Tovote *et al*. (2016).

As a functional interface between the limbic structures essential to survival defence behaviours, such as the central nucleus of the amygdala, the hypothalamus and medial prefrontal cortex (An *et al*. 1998; Petrovich *et al*. 2001; Canteras, 2002; Gross & Canteras, 2012; LeDoux, 2012; Linnman *et al*. 2012), and the lower brainstem and spinal cord (Fig. 2), the PAG plays a major role in integrating responses to internal and external threats (Blanchard *et al*. 1981; Bandler *et al*. 2000; Sokolowski & Corbin, 2012) that maximise an animal's survival by generating a repertoire of conditioned and unconditioned fear behaviours (Bandler *et al*. 1985; Bandler & Depaulis, 1991; Bandler & Shipley, 1994; Depaulis *et al*. 1994; Chen *et al*. 2015; Deng *et al*. 2016; Tovote *et al*. 2016).

Survival behaviours orchestrated by the PAG can be categorised into active and passive coping strategies (Fig. 1). Active coping is evoked by activation of the dl/lPAG, whereas passive coping is triggered by activation of the ventrolateral column. Active coping strategies (e.g. confrontation, fight or flight) are evoked if the stressor is escapable (e.g. brief acute pain, close encounter with a predator). By contrast, passive coping strategies (e.g. quiescence, recuperation, freezing) are elicited if the stressor is inescapable (e.g. visceral pain, proximity to or capture by a predator), thus facilitating recovery and healing but also via identified dl/lPAG–vlPAG functional excitatory connections (Tovote *et al*. 2016), preparing the animal to react or escape when opportunity arises.

A key component of CNS strategies for effective defence behaviour is the top down modulation of sensory transmission in the spinal dorsal horn and, since the original description of ‘stimulation‐produced analgesia’ from the PAG (Reynolds, 1969), attention has focused on descending pain modulatory systems that originate in the brainstem and operate at the level of the spinal cord.

## Periaqueductal grey: role in survival

### Top down control of spinal sensory processing

The spinal dorsal horn is the location of the first synapse in pain pathways and the capacity for the PAG to filter out nociceptive transmission at this early stage has long been recognised as essential for the execution of survival behaviours, as it minimises nociceptor‐driven sensory distraction and motor disturbances that would otherwise perturb effective actions (Waters & Lumb, 1997, 2008).

In this context, we now know that the descending pain modulatory system that originates from d/dl‐ and vlPAG discriminates not only between spinal processing of low intensity mechanosensitive inputs *versus* high intensity (nociceptive) inputs but, importantly, between nociceptive inputs of different behavioural significance, i.e. those conveyed in A‐ *versus* C‐nociceptors (Waters & Lumb, 2008). In their 2008 paper, Waters and Lumb provided mechanistic evidence that differential control of A‐ *versus* C‐fibre‐evoked responses of dorsal horn neurones from both the ventrolateral and dorsolateral/lateral columns of the PAG results from the modulation of spinal segmental inhibition. From a behavioural perspective, A‐ and C‐fibre nociceptors convey different qualities of the nociceptive message; well‐localised, rapidly conducted, ‘pricking pain’ that is tolerable *versus* poorly localised ‘aching/burning pain’ that is slowly conducted and can be intolerable. It has been proposed that suppression of C‐fibre‐mediated pain and simultaneous enhancement of A‐fibre‐mediated pain has important consequences in relation to both active and passive coping strategies as it would filter out unwanted distracting information and leave intact, or even augment, the ‘useful’ component of the pain signal that can provide motivation and guidance (Leith *et al*. 2007; Waters & Lumb, 2008; Drake *et al*. 2016).

The hypothesis that descending control of spinal sensory processing acts to filter out distracting information *en route* to supraspinal motor control centres has, until now, been based on indirect evidence. Recently however, Cerminara and colleagues provided evidence that the PAG can modulate cerebellar responses to sensory inputs (Cerminara *et al*. 2009) and this finding was subsequently advanced and refined in studies of PAG influences on spinal processing of sensory input to pre‐cerebellar pathways.

**Figure 3:**
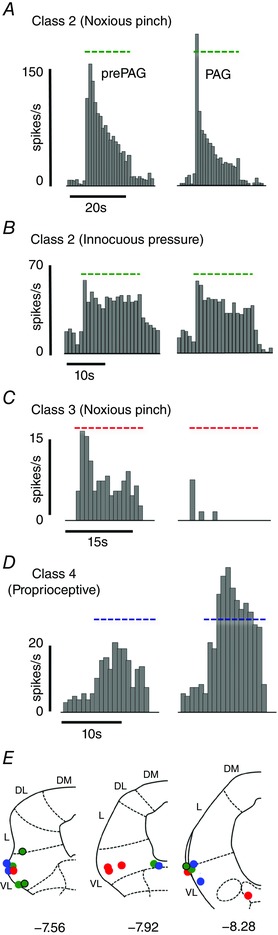
Periaqueductal grey selectively alters spino‐olivary neuronal responses to different qualities of sensory input *A*, typical example of the response of a class 2 neuron to noxious pinch (3.6 N): peristimulus time histogram (PSTH, spikes per 1 s bin) are shown before (pre‐PAG) and during (PAG) vlPAG chemical excitation with dl‐homocysteic acid in the anaesthetised rat. Dotted horizontal line in each of the PSTHs indicates the onset and duration of the peripheral stimulus. *B*, same as *A* except example of class 2 neuron response to innocuous pressure (0.5 N). *C*, same as *A* except example of class 3 neuron response to noxious pinch (3.6 N). *D*, same as *A* except example of class 4 neuron response to innocuous ankle joint manipulation. *E*, standard transverse maps of the left PAG at three rostrocaudal levels to show histological reconstruction of injection sites. Coordinates are relative to bregma. DL, dorsolateral; DM, dorsomedial; L, lateral; VL, ventrolateral. Green indicates class 2 (noxious pinch); green with black outline, class 2 (noxious pinch and innocuous pressure); red, class 3; blue, class 4. Adapted with permission from Koutsikou *et al*. (2015).

Motor behaviours essential for survival are guided not only by information about the *external* environment that is provided by cutaneous mechanosensitive and A‐fibre nociceptive inputs, but also by information from the *internal* environment, and in particular proprioceptive information that signals body position and movement. In this context, our recent studies have made the important finding that the PAG enhances proprioceptive transmission in spino‐olivary pathways (Fig. 3 *D*), which in turn forward information to the cerebellum (Fig. 3). The cerebellum is the largest sensorimotor structure in the brain and, as such, behaviourally relevant selectivity in descending control, including enhancement of responses to proprioceptive inputs, demonstrates the capacity of the PAG to regulate sensory input to supraspinal motor control centres and, as a consequence, to ensure the coordination of appropriate defensive behaviours in aversive situations when the PAG becomes active.

It is clear that the PAG can selectively control sensory input to the brain that ensures the execution of appropriate behaviours, but until recently, the question of whether the PAG has direct effects on the motor apparatus that drives behaviour remained unanswered.

### Top down control of motor circuits

Descending control from the PAG is essential (Keay & Bandler, 2001) to elicit motor responses characteristic of survival behaviours. However, little is known of the neural circuits that mediate the diversity of behavioural responses associated with PAG activation (Fig. 1). This is a significant gap in our understanding given the survival importance of initiating, adapting and maintaining coordinated motor responses in aversive and threatening situations.

To elicit active or passive motor responses, the PAG must engage ultimately with spinal motor circuits and, importantly, we have recently reported facilitation of α‐motoneurone excitability from the vlPAG (Koutsikou *et al*. 2014). These effects could be mediated by direct projections from the PAG to the spinal ventral horn (Mouton & Holstege, 1994) and/or indirectly (Mantyh, 1983; Tovote *et al*. 2016), including via the cerebellum (Dietrichs, 1983; Sillery *et al*. 2005). A cerebellar link is supported by the identification of anatomical connections between the PAG and pre‐motor structures such as the lateral reticular nucleus (Roste *et al*. 1985) and the inferior olive, the sole source of climbing fibres to the cerebellum (Swenson & Castro, 1983a,b; Rutherford *et al*. 1984; Watson *et al*. 2013; Koutsikou *et al*. 2014). Importantly, a powerful physiological link between the PAG and the cerebellum has been identified electrophysiologically (Koutsikou *et al*. 2014).

Unequivocal evidence for a top down PAG‐cerebellar link in the control of spinal motor outflow and defence behaviour is provided by reports that (i) powerful descending facilitatory influences from the vlPAG on spinal α‐motoneurone excitability is abolished by cerebellectomy, and (ii) targeted lesions of cerebellar input–output pathways abolishes the vlPAG‐induced increase in α‐motoneurone excitability and disrupts innate and fear‐conditioned freezing behaviour (Koutsikou *et al*. 2014).

However, it is not known how pathways from the PAG to the spinal cord that are direct and indirect (such as those that involve the cerebellum) might interact to co‐ordinate motor outflow.

In addition to identifying novel PAG circuits with supraspinal motor structures, our findings on top down control of motor function at the level of the spinal cord may also generate discussion. This is because, at first sight, vlPAG‐evoked increases in muscle tone appear at odds with the classic view that the vlPAG coordinates *passive* coping strategies, which are associated with quiescence and withdrawal from the environment. However, fear evoked freezing from the vlPAG is well documented and, to maintain a tense posture, requires increased muscle tone as reported by Misslin (2003). Furthermore, an effect of vlPAG on α‐motoneurone excitability is at odds with the hypothesis that increases in muscle tone seen in fear‐induced freezing behaviours is mediated by intra‐PAG inhibitory mechanisms regulated by the ventrolateral sector (Walker & Carrive, 2003), rather than by effects of the ventrolateral sector on motor outflow as suggested by our recent studies (Koutsikou *et al*. 2014; Koutsikou *et al*. 2015) and those of Tovote *et al*. (2016).

### Top down integration of sensory and motor systems

**Figure 4:**
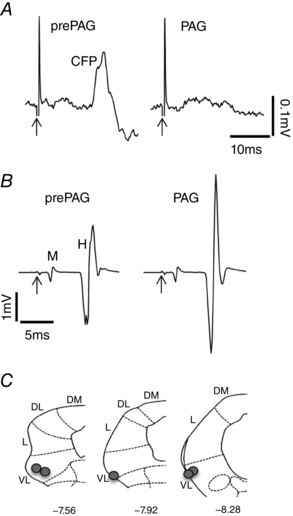
Periaqueductal grey activation results in simultaneous modulation of (*A*) ascending transmission to the cerebellum and (*B*) spinal motor circuits *A*, example of averaged climbing fibre field potentials (CFPs) recorded from the surface of the cerebellar cortex in the anaesthetized rat (C1 zone of left copula pyramidis). *B*, examples of averaged M‐wave (M) and H‐reflex (H) responses, the latter indicative of α‐motoneurone excitability, recorded from the left plantaris muscle at the same time as *A*. All responses were evoked by electrical stimulation of the ipsilateral tibial nerve (< 1 mA). Each averaged example consists of five consecutive responses before (prePAG) and during (PAG) vlPAG chemical excitation with dl‐homocysteic acid (DLH). Arrows indicate onset of the electrical stimulus. *C*, standard transverse maps of the left PAG to show injection sites of DLH in the vlPAG (filled circles), from which the effects of vlPAG activation on peak‐to‐peak amplitude of M‐wave and H‐reflex and CFP were tested. The coordinates are relative to bregma (DL, dorsolateral; DM, dorsomedial; L, lateral; VL, ventrolateral). Adapted with permission from Koutsikou *et al*. (2015).

To date, data generated in separate studies provide clear evidence that the vlPAG is the source of top down control of either sensory processing or motor outflow. Importantly, in order for the vlPAG to execute its survival role effectively requires that neurones in this region are able to integrate sensory and motor functions in a coordinated way, as evidenced by our recent findings (Koutsikou *et al*. 2015). In these studies, neuronal stimulation at individual sites in the vlPAG resulted in suppression of cerebellar sensory transmission (inhibition of peripheral nerve‐evoked cerebellar cortical field potentials), accompanied simultaneously by facilitation of spinal motor outflow (increased α‐motoneurone excitability, Fig. 4), providing evidence that the PAG acts on both sensory and motor systems simultaneously.

## Concluding remarks

As the PAG is the gatekeeper of spinal sensory transmission during aversive behaviour, its ability to exert selective control over sensory information of different modalities, and of different behavioural significance, including that transmitted to motor control centres, is pivotal to its role in the coordination of behaviours essential for survival. Importantly, it is now evident that selective control of sensory processing may be part of an integrated system whereby the PAG can orchestrate sensory and motor functions thus enabling behaviours to be executed with the appropriate degree of precision and strength, thereby assisting survival.

## Additional information

### Competing interests

We confirm that the authors have no conflicts of interest.

### Author contributions

Experimental work was carried out in the laboratories of B.M.L. and RA, School of Physiology, Pharmacology & Neuroscience at University of Bristol. Conception or design of the work: S.K., B.M.L., R.A.; acquisition, analysis, or interpretation of data for the work: S.K.; drafting the work or revising it critically for important intellectual content: S.K., B.M.L., R.A. All authors have approved the final version of the manuscript and agree to be accountable for all aspects of the work. All persons designated as authors qualify for authorship, and all those who qualify for authorship are listed.

### Funding

Biotechnology and Biological Sciences Research Council (BBSRC) BB/G012717/1.
